# Weak Interlayer Interaction in 2D Anisotropic GeSe_2_

**DOI:** 10.1002/advs.201801810

**Published:** 2018-12-20

**Authors:** Yusi Yang, Xia Wang, Shun‐Chang Liu, Zongbao Li, Zhaoyang Sun, Chunguang Hu, Ding‐Jiang Xue, Gengmin Zhang, Jin‐Song Hu

**Affiliations:** ^1^ Key Laboratory for the Physics and Chemistry of Nanodevices and Department of Electronics Peking University Beijing 100871 China; ^2^ Beijing National Laboratory for Molecular Sciences (BNLMS) CAS Key Laboratory of Molecular Nanostructure and Nanotechnology CAS Research/Education Center for Excellence in Molecule Science Institute of Chemistry Chinese Academy of Science Beijing 100190 China; ^3^ School of Material and Chemical Engineering Tongren University Tongren 554300 China; ^4^ HuSchool of Chemical Sciences University of the Chinese Academy of Sciences Beijing 100049 China; ^5^ State Key Laboratory of Precision Measuring Technology and Instruments Tianjin University Tianjin 300072 China

**Keywords:** binding energy, cleavage energy, germanium diselenide, interlayer interactions, translation energy

## Abstract

Germanium diselenide (GeSe_2_) has recently emerged as a new member of in‐plane anisotropic 2D materials, notable for its wide bandgap of 2.74 eV, excellent air stability, and high performance in polarization‐sensitive photodetection. However, the interlayer interaction in GeSe_2_ has never been reported, which usually plays an important role in layer‐number‐dependent physical properties. Here, the interlayer coupling in GeSe_2_ is systematically investigated from theory to experiment. Unexpectedly, all of density functional theory (DFT) calculations about layer‐dependent band structures, cleavage energy, binding energy, translation energy, and interlayer differential charge density demonstrate the much weaker interlayer interaction in GeSe_2_ when compared with black phosphorus (BP). Furthermore, both thickness‐dependent and temperature‐dependent Raman spectra of GeSe_2_ flakes, which exhibit no detectable changes of Raman peaks with the increase in thickness and a small first‐order temperature coefficient of −0.0095 cm^−1^ K^−1^, respectively, experimentally confirm the weakly coupled layers in GeSe_2_. The results establish GeSe_2_ as an unusual member of in‐plane anisotropic 2D materials with weak interlayer interaction.

## 1 Introduction

The interlayer interaction in 2D‐layered materials attracts increasing interest as an efficient tool for controlling the optical, electronic, thermal, vibrational, and mechanical properties of 2D materials through precise modulation of the number of layers.^1^, ^2^, ^3^ For example, MoS_2_ undergoes a crossover from indirect bandgap in the bulk or multilayers to direct bandgap in monolayer limit, enabling MoS_2_ to absorb and emit light efficiently.^3^, ^4^, ^5^ In particular, the recently isolated black phosphorus (BP) with a unique in‐plane anisotropy demonstrates an exceptional tunability of its bandgap ranging from 0.3 eV for bulk to 2.0 eV for monolayer due to its strong interlayer interaction explained by quantum confinement in out‐of‐plane direction,^6^, ^7^, ^8^, ^9^, ^10^ making it especially suitable for optoelectronic applications such as photodetection ranging from far‐infrared to visible regime.^9^, ^11^

Nevertheless, when fabricating practical devices, the aforementioned benefits of tunability of 2D materials are partly counterbalanced by the following issues: i) The strong interlayer interaction in 2D materials often results in unavoidable electronic packaging with foreign substrate described by substrate interaction, leading to large variation in electronic properties of monolayer and thereby affecting its applications in nanoelectronics.^12^ For instance, MoS_2_ exhibits transition from direct to indirect bandgap when the monolayer is stacked to multilayer structures, seriously restricting its optoelectronic applications.^3^, ^5^ ii) It is still a well‐recognized challenge to prepare large‐area monolayers or multilayers with explicitly specified number of layers, severely hindering the further optimization of device performance.

Therefore, it is urgent to explore new 2D materials that can stabilize the novel properties arising from the monolayer while in the bulk form. Fortunately, there is a candidate material exhibiting monolayer behavior in its bulk form, rhenium disulfide (ReS_2_), attributed to its particularly weak interlayer interaction originating from the Peierls distortion of the 1T crystal structure.^13^, ^14^, ^15^ ReS_2_ shows layer‐independent electronic, optical, and vibrational properties, in contrast to other 2D materials explored to date.^13^ This offers exciting prospect to explore the 2D physics in 3D form without the preparation of monolayers. However, the exploration of such weak interlayer interaction in 2D materials is still in its initial stage due to the fact that the corresponding studies mainly focus on ReS_2_, as well as its counterpart ReSe_2_.^16^

This work presents a new member of 2D materials with weak interlayer interaction, germanium diselenide (GeSe_2_), a recent addition to the in‐plane anisotropic 2D materials family,^17^ which is further demonstrated by azimuth‐dependent reflectance difference microscopy (ADRDM) in this study. Then, first‐principle calculations based on density functional theory (DFT) are applied to systematically study the weak interlayer interaction in GeSe_2_ through layer‐dependent band structures, cleavage energy, binding energy, translation energy, and interlayer differential charge density, respectively. Finally, thickness‐dependent and temperature‐dependent spectra of GeSe_2_ flakes are obtained to further confirm the weak interlayer coupling in GeSe_2_ from experiment. The combined theoretical and experimental investigation demonstrates that GeSe_2_ can be a new candidate for in‐plane anisotropic 2D materials with weak interlayer interaction.

## 2 Results and Discussion

Before studying the interlayer interaction in GeSe_2_, we first investigated the in‐plane anisotropy of GeSe_2_ by ADRDM, which was a nondestructive, surface‐sensitive, rapid and directly visualizing detection technique to characterize the optical anisotropy of the sample. The detection principle of ADRDM was to directly measure the normalized difference in reflectance (Δ*R*) between two arbitrary orthogonal directions in the surface plane (*a* and *b*) when the sample is irradiated by incident polarized light, which can be defined as^18^

(1) $$\frac{\Delta R}{R}=2\frac{R_{a}-R_{b}}{R_{a}+R_{b}}=2N$$

where *R*_a_ and *R*_b_ are the intensities of reflectance along *a* and *b* directions. The dimensionless value N(θ) alters as incident direction of linearly polarized light changes, which can be described as^18^, ^19^, ^20^

(2) $$N\left(\theta \right)=\frac{R_{x}-R_{y}}{R_{x}+R_{y}}\cos2\left(\theta -\theta_{0}\right)$$

where θ represents the angle between incident polarized light and *x* axis of GeSe_2_. Therefore, from the above equation, we could see that the value of N(θ) can be varied periodically with the changing azimuth angle of incident polarized light toward materials with in‐plane anisotropy. In particular, ADRDM can collect N(θ) at all pixels and thus be used as an imaging tool, offering a direct visualization of the anisotropic contrast.

**Figure 1:**
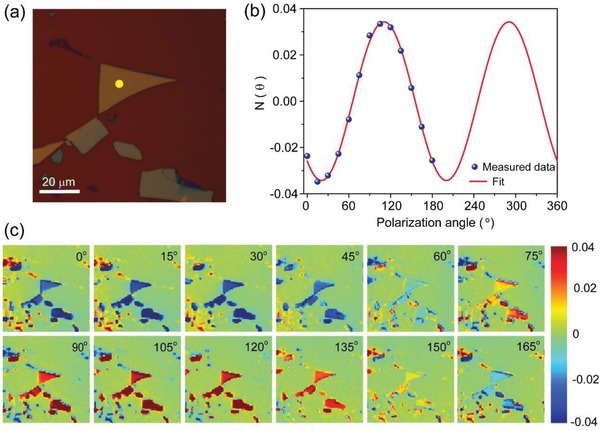
a) Optical image of GeSe_2_ flakes transferred onto a SiO_2_/Si substrate by mechanical exfoliation. b) ADRDM results of GeSe_2_ flake in the region marked with a yellow spot in (a). c) ADRDM images of GeSe_2_ flakes as function of rotation angle.

In the measurement, we rotated the incident polarization angle at a step of 15° while kept the sample still. GeSe_2_ thin flakes were mechanically exfoliated from bulk GeSe_2_ crystal (Figures S1 and S2, Supporting Information). The ADRDM results of a GeSe_2_ flake exfoliated on an isotropic SiO_2_/Si substrate were shown in **Figure** **1**. According to Figure 1b, it can be easily acquired that N(θ) exhibited obvious change under different incident angles, and the value of N(θ) was maximized at ≈110° whereas it was minimized at 20°. It was clear that the relationship between N(θ) and incident angle can be well fitted with Equation (2). Figure 1c presented ADRDM images recorded at varied angles. The intensity of N(θ) on the GeSe_2_ flakes reached a minimum value at 15°–30° (dark blue) and a maximum value at 105°–120° (dark red) while the collected signals of the isotropic SiO_2_/Si substrate remained zero (green) for all angles. Therefore, the ADRDM measurement forcefully confirms the in‐plane anisotropy of GeSe_2_, consistent with previously reported results from other measurements such as angle‐resolved polarized Raman spectroscopy.^17^, ^19^

**Figure 2:**
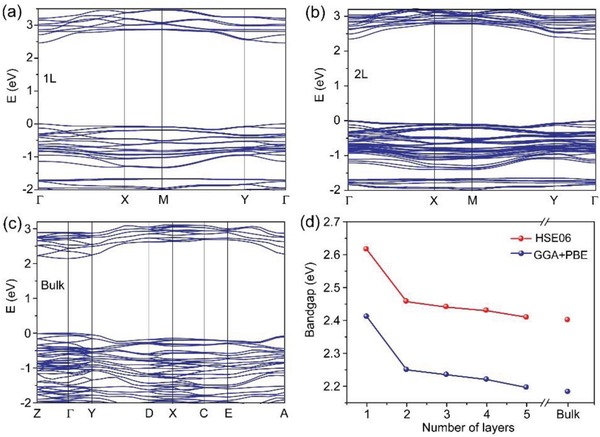
DFT calculated electronic band structure of a) monolayer, b) bilayer, and c) bulk GeSe_2_ with the GGA‐PBE functional. d) Evolution of bandgap as a function of the number of layers with the HSE06 and GGA‐PBE methods, respectively.

When considering the interlayer interaction, anisotropic 2D materials can be divided into two categories, including strong interlayer coupling such as BP, and weak interlayer interaction such as ReS_2_. To systematically investigate the interlayer interaction of GeSe_2_, we first performed first‐principle calculations based on DFT to calculate the electronic band structures and bandgap evolution of monolayer‐to‐bulk GeSe_2_ by using generalized gradient approximation of Perdew–Burke–Ernzerhof (PBE) (GGA‐PBE) functional. As shown in **Figure** **2**a–c, all of the monolayer, bilayer, and bulk GeSe_2_ had a direct bandgap with a conduction band minimum (CBM) and a valence band maximum (VBM) at Γ‐point without a direct‐to‐indirect bandgap transition. Meanwhile, due to the existence of bandgap underestimation in GGA‐PBE functional, we also employed the hybrid functional approximation using HSE06 exchange‐correlation term to calculate a more accurate bandgap for comparison. Figure 2d presented the bandgap evolution as a function of the number of layers. It was obvious that there was only a minor bandgap shortening from the monolayer to bulk GeSe_2_ by both of the GGA‐PBE and HSE06 methods. Therefore, our calculated results indicate that the interlayer interaction in GeSe_2_ could be too weak to alter the electronic band structures.

**Figure 3:**
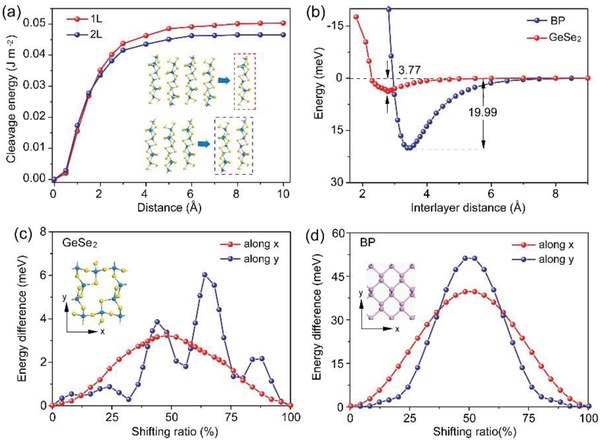
a) Cleavage energy as a function of the separation distance between a monolayer/bilayer and its neighboring four‐layer/trilayer, respectively. b) Binding energy of bilayer GeSe_2_ and BP as a function of the interlayer spacing. c) In‐plane translation energy of GeSe_2_ along *x* and *y* directions. d) In‐plane translation energy of BP along *x* and *y* directions.

Although we have reported the preparation of GeSe_2_ thin layers through the conventional mechanical exfoliation via scotch tape,^17^ the corresponding cleavage energy is still unclear so far. To evaluate the cleavage energy, we calculated the separation of a monolayer GeSe_2_ from a neighboring four‐layer (red inset of **Figure** **3**a), in which four layers were fixed serving as a model of the bulk while the monolayer was flexible. The separation distance in the equilibrium geometry was defined as zero. The total energy with increasing the separation distance between the monolayer and four‐layer was calculated to simulate the exfoliation process. Figure 3a (red curve) showed the resulting cleavage energy as a function of the separation distance, which gradually increased with the separation distance enlarging and finally congregates in a constant value of ≈0.05 J m^−2^. In addition, we also simulated the separation of a GeSe_2_ bilayer from a neighboring trilayer (blue inset of Figure 3a) for comparison. The corresponding cleave energy was ≈0.046 J m^−2^, nearly identical to the monolayer cleavage case. The calculated cleavage energy of GeSe_2_ was far smaller than that of many other 2D materials, such as experimentally estimated value of graphene (0.37 J m^−2^),^20^ and DFT calculated value of GeP_3_ (1.14 J m^−2^),^21^ GeS (0.52 J m^−2^),^22^ and NaSnP (0.81 J m^−2^),^23^ clearly demonstrating the ultraweak interlayer interaction in GeSe_2_.

To further study the interlayer binding in GeSe_2_ from different aspects, we calculated the binding energy of GeSe_2_ as a function of the interlayer spacing in bilayer GeSe_2_, while choosing anisotropic BP with well‐known strong interlayer interaction as a comparison. As shown in Figure 3b, the binding energy of the bilayer GeSe_2_ was ≈3.77 meV, less than that of BP (≈19.99 meV), illustrating that GeSe_2_ bilayers were weakly bonded to each other compared with BP. Then, we turned to evaluate the interlayer binding of GeSe_2_ through calculating the in‐plane translation energy, which could be defined as the energy difference between the different stacking positions. It was clear that shifting one GeSe_2_ monolayer over another did not lead to any significant change in total energy; it was only ≈6.37 meV along *x* direction and ≈11.94 meV along *y* direction, respectively (Figure 3c). For comparison, analogous calculation of translating one BP monolayer over the other one along in‐plane *x* and *y* directions obtained the translating energy up to 80.16 and 102.71 meV (Figure 3d), thus giving us another indication that the interlayer interaction was very weak for adjacent GeSe_2_ layers, compared with that of BP. In brief, the above calculated cleavage energy, binding energy, and translation energy all demonstrate weak interlayer coupling in GeSe_2_.

**Figure 4:**
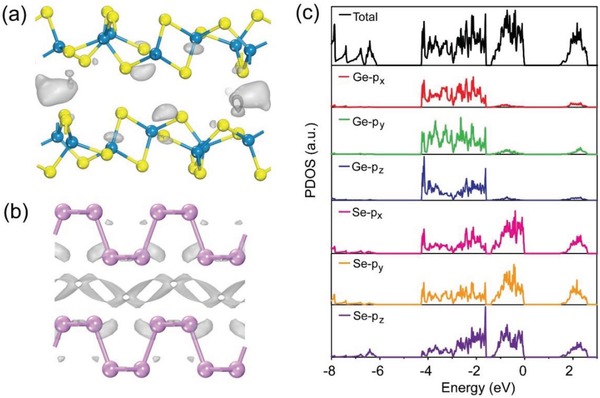
Interlayer differential charge density of a) GeSe_2_ and b) BP, respectively. c) Partial density of states (PDOS) of GeSe_2_.

A more direct way to investigate the character of interlayer interaction is the interlayer differential charge density, which can be induced through assembling the bulk system from isolated monolayers, thereby representing the charge redistribution during this accumulating process. The corresponding interlayer density difference was visualized in **Figure** **4**. It was obvious that negligible charge was distributed in the interlayer region of GeSe_2_ bilayers (Figure 4a), illustrating almost no covalency, while there was a significant number of electrons localized in the interlayer region of BP with partial covalent characteristic (Figure 4b). Therefore, we can conclude that the ultraweak interlayer interaction in GeSe_2_ was attributed to the insufficient bonding charges between the bilayers. Moreover, based on the crystal structure of monolayer GeSe_2_ (Figure 4a), where Ge atoms were sandwiched by Se atoms, the interlayer interaction in GeSe_2_ might be from the coupling of p_z_ orbitals of interlayer Se atoms. Thus, to deeply explain the weak interaction between adjacent atom layers in GeSe_2_, we further calculated the partial density of states (PDOS) of GeSe_2_. As shown in Figure 4c, it can be readily obtained that the p_z_ orbital of Se atom mainly participated in intralayer hybridization, thus resulting in the weak interlayer coupling between two adjacent layers and then the layer‐number‐independent physical properties.

**Figure 5:**
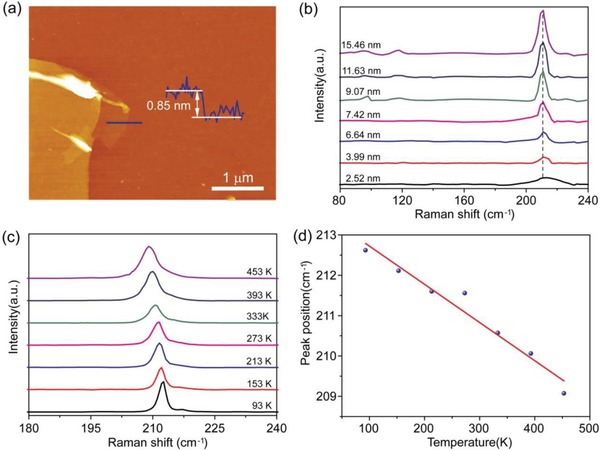
a) AFM image of monolayer GeSe_2_. b) Raman spectra of GeSe_2_ flakes with different thickness. c) Raman spectra of GeSe_2_ flake at temperatures ranging from 93 to 453 K. d) Temperature dependence of the peak position of the Ag mode at 210 cm^−1^.

To experimentally study the interlayer interaction in GeSe_2_, we now carefully analyze the Raman shifts in Raman spectra of GeSe_2_ thin flakes with different thicknesses, a conventional method to detect the interlayer coupling. GeSe_2_ flakes were mechanically exfoliated from bulk crystal and monolayer GeSe_2_ was characterized by atomic force microscopy (AFM), as shown in **Figure** **5**a and Figure S4 in the Supporting Information. Then, Raman shifts in Raman spectra of GeSe_2_ thin flakes with different thicknesses were investigated (Figure S5, Supporting Information). According to Figure 5b, there are nearly no detectable changes with the increase in thickness, probably induced by weak interlayer coupling in GeSe_2_. In contrast, the widely explored anisotropic 2D BP demonstrated a highly sensitive variation in the Raman peak positions with different number of layers.^24^

In addition to the room temperature Raman spectroscopy, we also performed the temperature‐dependent Raman spectroscopy, which can provide exact information on thermal expansion, thermal conductivity, and interlayer coupling.^25^, ^26^, ^27^, ^28^ In view of convenience, we selected the most intensive Ag mode located at 210 cm^−1^ for further detailed analysis. The thickness of GeSe_2_ flake was first characterized as 55.6 nm by AFM measurement (Figure S6, Supporting Information). As shown in Figure 5c, the Raman peaks exhibited a red shift when the temperature increased from 93 to 453 K. This observed shift of the Raman peak position with temperature can be well explained through anharmonic effects developed by Balkanski et al.,^29^ which is based on the phenomenon of the optical phonon decay into two (three phonon process) or three (four phonon process) acoustic phonons with equal energies arising from lattice potential cubic and quartic anharmonicity.^30^ Therefore, the variation of Raman frequency with temperature, Δω (*T*), can be described by

(3) $$\Delta \omega \left(T\right)=A\left[1+\frac{2}{e^{x}-1}\right]+B\left[1+\frac{3}{e^{y}-1}+\frac{3}{{\left(e^{y}-1\right)}^{2}}\right]$$

where *x* = ℏω_0_/2*k*_b_ *T*, *y* = ℏω_0_/2*k*_b_ *T*, ℏ is Planck's constant divided by 2π, *k*_b_ is the Boltzmann constant, and *A* as well as *B* are anharmonic constants.^30^ Figure 5c showed the Raman peak position shift in Ag mode (210 cm^−1^) as a function of temperature, demonstrating an obvious linear dependence with increasing temperature, which can be depicted by a first‐order temperature coefficient (χ) according to a linear equation

(4) $$\omega \left(T\right)=\omega_{0}+\chi T$$

where ω_0_ is the phonon frequency of the mode at 0 K and χ is the first‐order temperature coefficient.^25^, ^31^ The extracted χ for mode Ag at 210 cm^−1^ is −0.0095 cm^−1^ K^−1^. The obtained value for GeSe_2_ is much smaller than that for other 2D layered materials such as BP (−0.023 cm^−1^ K^−1^ for A^2^_g_ mode),^32^ SnS (−0.036 cm^−1^ K^−1^ for A^2^_g_ mode),^33^ and SnSe (−0.0377 cm^−1^ K^−1^ for A^2^_g_ mode),^27^ while nearly equal to ReSe_2_ (−0.0074 cm^−1^ K^−1^ for A_g_‐like mode)^34^ and in good agreement with the previous report.^19^ It was reported that the first‐order temperature coefficients of the Raman active modes were associated with the interlayer interaction between the layers of 2D layered materials.^34^ For example, the strong van der Waals interaction in BP, SnS, and SnSe led to their large first‐order temperature coefficients. In contrast, the smaller first‐order temperature coefficient of GeSe_2_, comparable to that of ReSe_2_ with well‐known weak interlayer coupling, clearly indicated the weaker interlayer interaction in GeSe_2_. Therefore, we would not need to control the specific number of layers of GeSe_2_ in practical optoelectronic applications while making it easier for intercalation of ions.^35^ Briefly, both the room temperature thickness‐dependent and temperature‐dependent Raman spectra suggest that adjacent layers in GeSe_2_ are weakly coupled to each other, highly consistent with our theoretical predications.

## 3 Conclusion

In summary, we have systematically studied the interlayer interaction in GeSe_2_ from theory to experiment. ADRDM measurement clearly indicated the in‐plane optical anisotropy of GeSe_2_. DFT calculations about layer‐dependent band structures, cleavage energy, binding energy, translation energy, and interlayer differential charge density demonstrated the weak interlayer interaction in GeSe_2_. Room temperature thickness‐dependent and temperature‐dependent Raman spectra of GeSe_2_ flakes, which exhibited no detectable changes of Raman peaks with the increase in thickness and a small first‐order temperature coefficient of −0.0095 cm^−1^ K^−1^, respectively, further verified the weak interlayer coupling in GeSe_2_ from experiment, highly consistent with our theoretical predications. Our combined theoretical and experimental results introduce a new member of in‐plane anisotropic 2D materials with weak interlayer interaction.

## Conflict of Interest

The authors declare no conflict of interest.

## Supporting information

Supplementary
