# Supplementary material for: Weak Interlayer Interaction in 2D Anisotropic GeSe2
Source: Adv Sci (Weinh). 2018 Dec 20;6(5):1801810. doi: 10.1002/advs.201801810 (PMC6402401; doi:10.1002/advs.201801810)
Supplement: Supplementary file 1 — Supplementary [file ADVS-6-1801810-s001.pdf]

Copyright WILEY-VCH Verlag GmbH & Co. KGaA, 69469 Weinheim, Germany, 2018.

Supporting Information

for *Adv. Sci.,* DOI: 10.1002/advs.201801810

Weak Interlayer Interaction in 2D Anisotropic $GeSe_2$

Yusi Yang, Xia Wang, Shun-Chang Liu, Zongbao Li, Zhaoyang Sun, Chunguang Hu, Ding-Jiang Xue, Gengmin Zhang,* and Jin-Song Hu**

Copyright WILEY-VCH Verlag GmbH & Co. KGaA, 69469 Weinheim, Germany, 2016.

Supporting Information

Weak Interlayer Interaction in 2D Anisotropic $GeSe_2$

Yusi Yang, Xia Wang, Shun-Chang Liu, Zongbao Li, Zhaoyang Sun, Chunguang Hu, Ding-Jiang Xue,* Gengmin Zhang,* and Jin-Song Hu*

Y. Yang, Prof. G. Zhang*
Key Laboratory for the Physics and Chemistry of Nanodevices and Department of Electronics, Peking University, Beijing, 100871, China.
E-mail: zgmin@pku.edu.cn

Y. Yang, S.-C. Liu, Prof. D.-J. Xue*, Prof. J.-S. Hu*
Beijing National Laboratory for Molecular Sciences (BNLMS), CAS Key Laboratory of Molecular Nanostructure and Nanotechnology, CAS Research/Education Center for Excellence in Molecule Science, Institute of Chemistry, Chinese Academy of Science, Beijing,100190, China
E-mail: djxue@iccas.ac.cn, hujs@iccas.ac.cn

Prof. X. Wang, Prof. Z. Li
School of Material and Chemical Engineering, Tongren University, Tongren, 554300, China

Z. Sun, Prof. C. Hu
State Key Laboratory of Precision Measuring Technology and Instruments, Tianjin University, Tianjin, 300072, China.

S.-C. Liu, Prof. D.-J. Xue, Prof. J.-S. Hu
University of the Chinese Academy of Sciences, Beijing, 100049, China.

This file includes Experiment Section and Figure S1-S7.

Experimental Section

ADRDM measurement

A $GeSe_2$ flake was exfoliated onto a SiO_2/Si substrate. In the measurement, a polarizer and a liquid crystal variable retarder was rotated at a step of 15° to change the direction of incident polarized light. A CCD was used to record the ADRDM images of $GeSe_2$.

Raman measurement

Raman signals of $GeSe_2$ flakes with different thicknesses were obtained by a Horiba LabRAM HR Evolution system equipped with a 532 nm laser. To determine the thickness, atomic force microscopy (AFM, Bruker Dimension Icon microscope) was adopted. Temperature-dependent Raman spectra were performed on the same Raman system by changing the temperature from 93 K to 453 K.

DFT calculations

We used the Vienna ab initio simulation package (VASP)[1-2] to perform first-principles calculations based on density function theory to study the electronic structure and equilibrium geometries in conjunction with general gradient approximation (GGA) with the Perdew-Burke-Ernzerhofer (PBE) formula[3]. The ultra-soft pseudopotential was used to describe the exchange-correlation effects and electron-ion interactions. An energy cutoff of 500 eV was set for the plane-wave basis while the Brillouin-Zone (BZ) integrals[4] was sampled by a 5×5×1 Monkhorst-Pack k-points set. A maximum force criterion of 0.02 eV/Å was used and the perpendicular force was set as 0.01 eV/Å for the intermediate image relaxation. We placed the layered structure in the xy plane and added a 20 Å vacuum region for the slab model to eliminate interaction between periodic images. Meanwhile, the hybrid functional approximation of Heyd–Scuseria–Ernzerhof (HSE06)[5] was applied to acquire more accurate band structures for the cases, respectively.

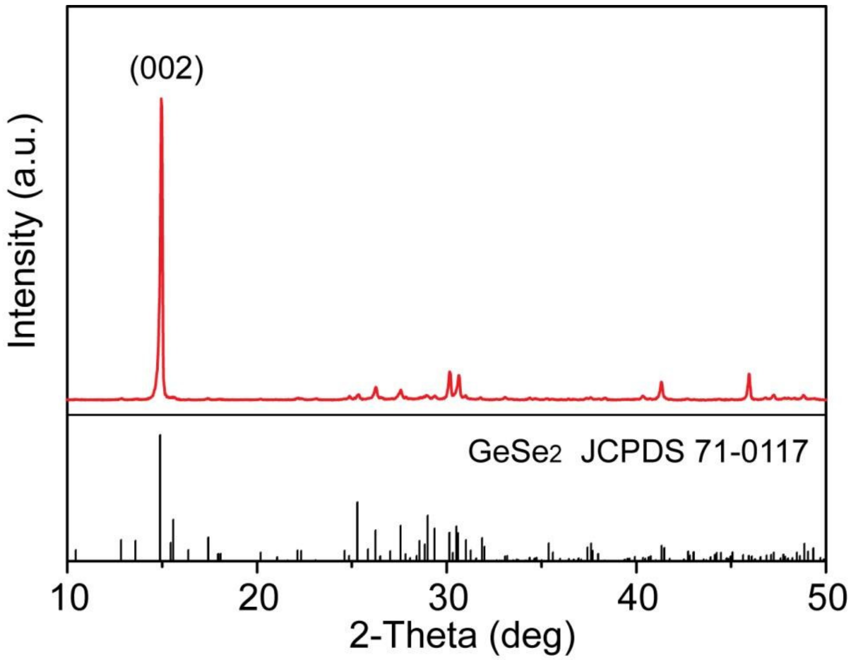

Figure S1. XRD pattern of $GeSe_2$ bulk material.

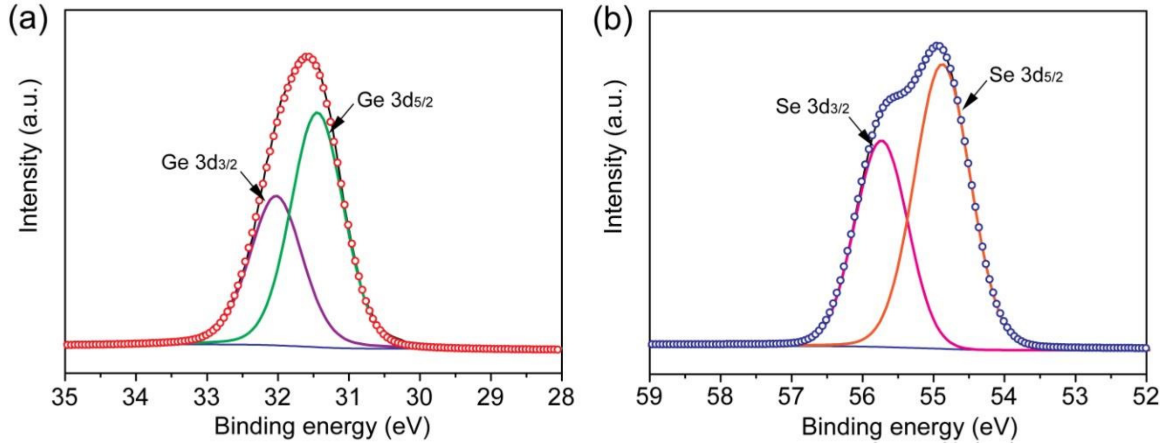

Figure S2. XPS spectra of (a) Ge 3d and (b) Se 3d in $GeSe_2$ bulk material.

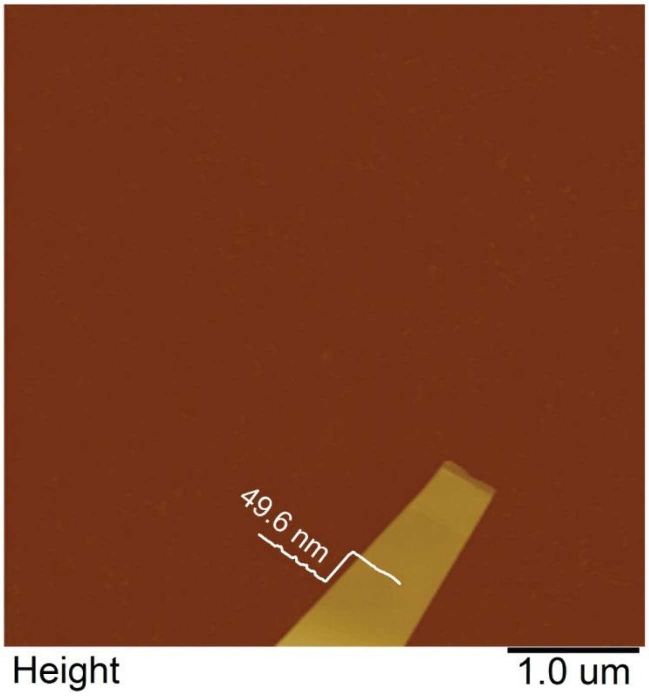

Figure S3. AFM image of $GeSe_2$ flake for ADRDM measurement.

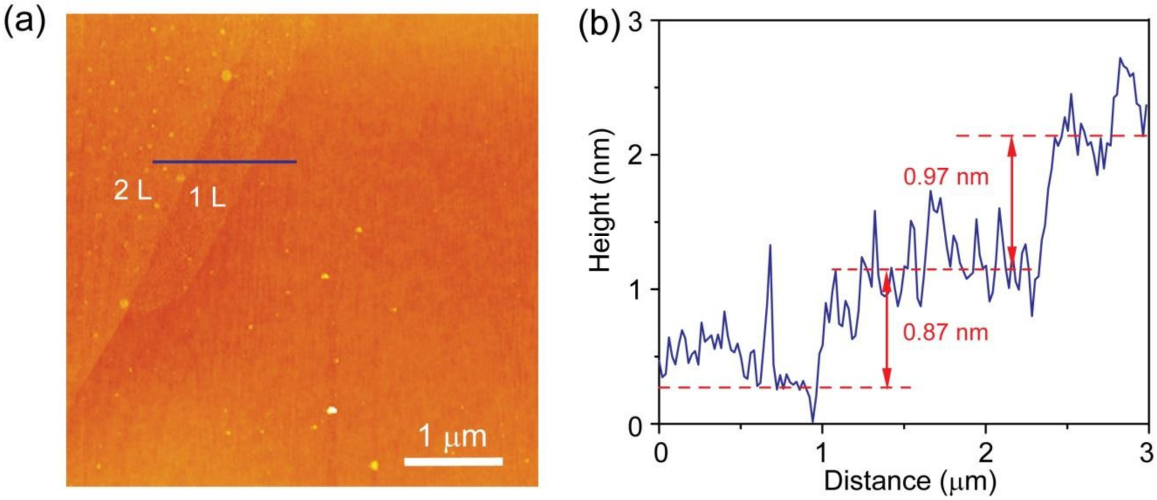

Figure S4. (a) AFM image of 1L and 2L $GeSe_2$. (b) Corresponding height profile.

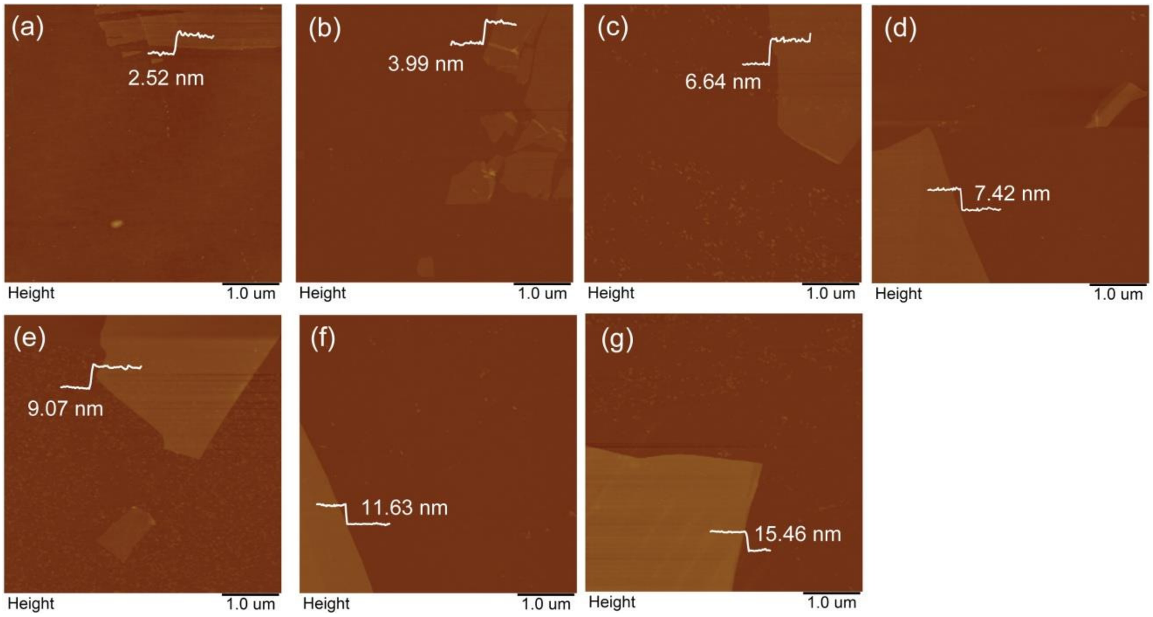

Figure S5. AFM images of $GeSe_2$ flakes with different thicknesses for Raman measurement.

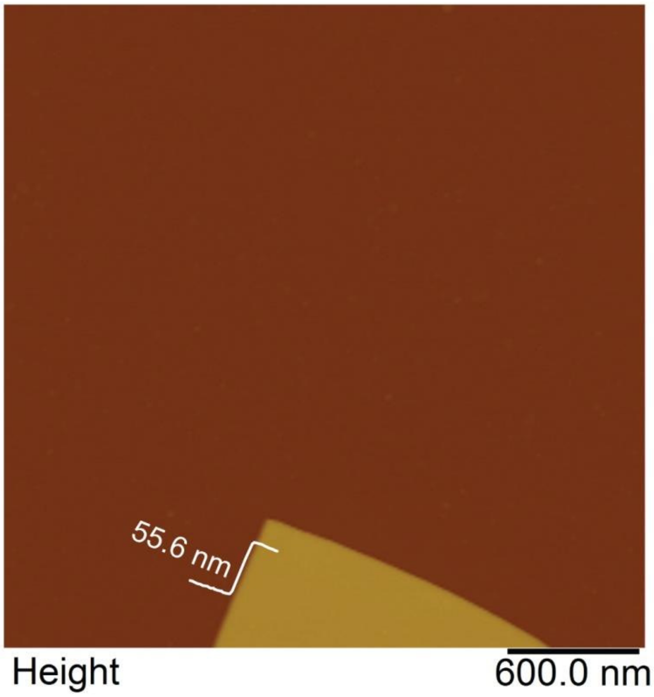

Figure S6. AFM image of $GeSe_2$ flake for temperature-dependent Raman measurement.

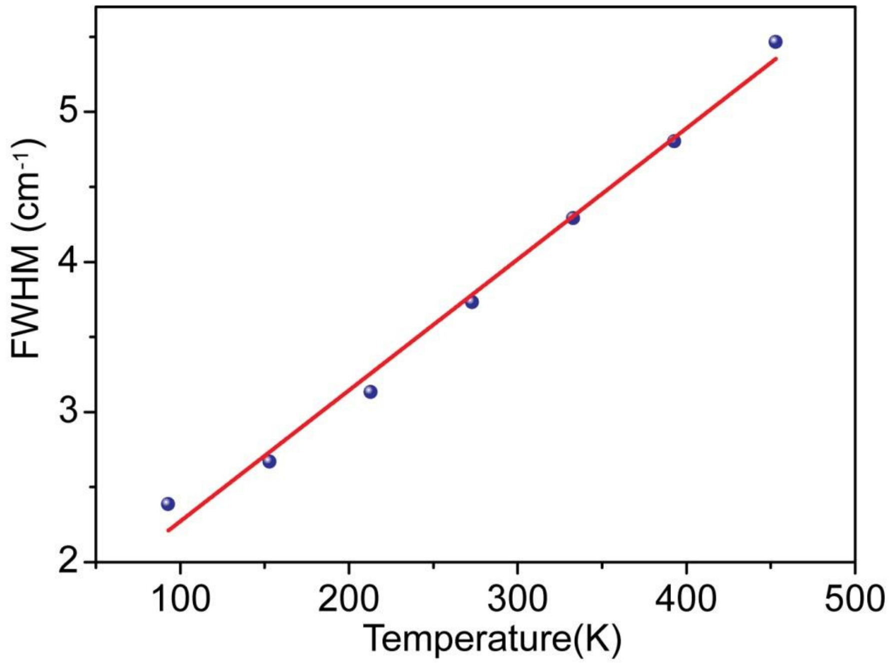

Figure S7. The FWHM values of the Raman mode at 210cm^{-1} under different temperatures.

References in Supporting Information

[1] G. Kresse, J. Hafner, Ab initio molecular-dynamics simulation of the liquid-metal amorphous-semiconductor transition in germanium. Phys, Rev. B. **1994**, 49, 14251-14269.

[2] G. Kresse, J. Furthermuller, Effect of Er doping on the electronic structure of optical properties of ZnO, Phys. Rev. B **1996**, 54, 11169-11186

[3] J.P. Perdew, K. Burke, M. Ernzerhof, Generalized Gradient Approximation Made Simple, Phys. Rev. Lett. **1996**, 77, 3865-3868.

[4] H.J. Monkhorst, J.D. Pack, Special points for Brillouin-zone integrations, Phys. Rev. B **1976**, 13, 5188-5192.

[5] J. Heyd, G. E. Scuseria and M. Ernzerhof, J. Chem. Phys. **2003**, 118, 8207–8215.
